# Validation of the Risk Instrument for Screening in the Community (*RISC*) among Older Adults in the Emergency Department

**DOI:** 10.3390/ijerph20043734

**Published:** 2023-02-20

**Authors:** Rónán O’Caoimh

**Affiliations:** 1 Department of Geriatric Medicine, Mercy University Hospital, Grenville Place, T12 WE28 Cork, Ireland; rocaoimh@hotmail.com; Tel.: +353-21-420-5976; 2 Clinical Research Facility Cork, Mercy University Hospital, University College Cork, T12 WE28 Cork, Ireland

**Keywords:** emergency department, frailty, screening, Clinical Frailty Scale, Risk Instrument for Screening in the Community, diagnostic accuracy, Identification of Seniors at Risk, PRISMA-7

## Abstract

Although several short-risk-prediction instruments are used in the emergency department (ED), there remains insufficient evidence to guide healthcare professionals on their use. The Risk Instrument for Screening in the Community (*RISC*) is an established screen comprising three Likert scales examining the risk of three adverse outcomes among community-dwelling older adults at one-year: institutionalisation, hospitalisation, and death, which are scored from one (rare/minimal) to five (certain/extreme) and combined into an *Overall RISC* score. In the present study, the *RISC* was externally validated by comparing it with different frailty screens to predict risk of hospitalisation (30-day readmission), prolonged length of stay (LOS), one-year mortality, and institutionalisation among 193 consecutive patients aged ≥70 attending a large university hospital ED in Western Ireland, assessed for frailty, determined by comprehensive geriatric assessment. The median LOS was 8 ± 9 days; 20% were re-admitted <30 days; 13.5% were institutionalised; 17% had died; and 60% (116/193) were frail. Based on the area under the ROC curve scores (AUC), the *Overall RISC* score had the greatest diagnostic accuracy for predicting one-year mortality and institutionalisation: AUC 0.77 (95% CI: 0.68–0.87) and 0.73 (95% CI: 0.64–0.82), respectively. None of the instruments were accurate in predicting 30-day readmission (AUC all <0.70). The *Overall RISC* score had good accuracy for identifying frailty (AUC 0.84). These results indicate that the RISC is an accurate risk-prediction instrument and frailty measure in the ED.

## 1. Introduction

Risk-prediction in healthcare is complex but important, particularly in an environment of increased demand with fixed or even diminishing resources available. Modelling risk is useful to improve the identification of individuals susceptible to adverse healthcare outcomes, which can in turn improve resource utilisation through the appropriate targeting of limited resources [1]. Risk modelling in healthcare is increasingly used in different settings, from emergency admissions to hospitals to care in the community [2]. However, it is not without challenges. The need for accurate risk-prediction in healthcare is exemplified by the rising pressure on already overcrowded emergency departments (ED) [3]. ED visits are increasing worldwide [4,5], with ED admissions accounting for a growing proportion of acute hospital bed days [6]. Older adults are the highest users of ED resources, often presenting with multiple and complex chronic conditions [7]. Older adults may be most affected by this rising demand, which results in overcrowding and increased waiting times [3].

Current demographic trends suggest that populations worldwide but especially in Europe are ageing, with an overall increasing disease burden [8]. With this comes an increased incidence [9] and high prevalence of frailty [10]. These changes have been accompanied by a greater proportion of older people with acute illness attending EDs globally, including in Ireland [3], where this study was conducted. Frailty is a complex age-associated syndrome characterised by vulnerability to physiological stressors, predisposing individuals to adverse healthcare outcomes [11]. Frailty is increasingly recognised as an emerging public health priority [12] and is associated with increased hospital length of stay (LOS), mortality, and healthcare costs [13,14,15]. Although frail older adults represent only approximately 10% of those attending ED [16], they represent the majority (60%) of older attendees [17] and have higher admission rates (ED conversion defined by the number of admissions as a proportion of attendees) [18]. The changing age profile of ED attendees is itself a public health concern. 

The relationship between risk of adverse events (i.e., risk level as determined by risk-prediction modelling) and frailty status remains unclear, and it is unknown if and to what extent the two constructs overlap [19,20]. Although no consensus definition for frailty is widely accepted [21], in clinical practice and research, operational definitions of frailty commonly incorporate the concept of risk of adverse outcomes [22]. Multiple frailty screening instruments are currently available, though few can be recommended for population-level screening [19]. Recent systematic reviews have identified that few risk-prediction instruments are available for use with community-dwelling older adults, and those that are available have limited diagnostic accuracy [19,23]. Similarly, there is insufficient evidence for short, usable, reliable, or validated risk-prediction tools for identifying risk of different health outcomes in the ED [24,25,26]. 

The Risk Instrument for Screening in the Community (*RISC*) [27] is a short, subjective, global, acceptable [28], and reliable [29] risk-prediction instrument for use by healthcare professionals. The *RISC* quickly screens and stratifies patients according to their one-year risk of three adverse outcomes (institutionalisation, hospitalisation, and death) based on a healthcare professional’s subjective scores on a five-point Likert scale, scored from one (rare/minimal) to five (certain/extreme) based on the healthcare professional’s judgement taking into account available information. The *RISC* correlates with a global measure of frailty in community dwellers [23], the Clinical Frailty Scale (CFS) [30], but has not been compared with more detailed frailty scales or an independent comprehensive geriatric assessment (CGA). Originally validated in a community setting, it is yet to be validated in ED. 

Given these points, this diagnostic-accuracy study aims to investigate the predictive validity of the *RISC* in detecting a selection of adverse outcomes among older people attending ED: hospitalisation (LOS and 30-day readmission rates) and one-year rates of institutionalisation and death (construct validity). The secondary objective is to measure the accuracy of the *RISC* to correctly identify and classify frailty status compared to a gold-standard battery of frailty assessment scales.

## 2. Materials and Methods 

### 2.1. Design and Patients

This study is a secondary analysis of an observational diagnostic accuracy study, the methods of which have been published elsewhere [20]. In summary, community-dwellers aged 70 years and over arriving to the ED of a tertiary referral university hospital in Ireland (Western) were eligible and were recruited using a consecutive sampling strategy. All those with a Manchester Triage System (MTS) score >1 (scale ranging from 1 to 5), indicating non-immediate priority [31], were considered for inclusion in the study. The MTS ranks patients according to five levels from low, i.e., five (non-urgent priority), through to high or level one (i.e., immediate priority) [31]. Persons in an unstable medical condition according to the MTS (i.e., a score of one), nursing home residents, and people admitted directly to intensive care or the cardiac care unit were excluded from the study. Those in long-term residential care were excluded given the high prevalence of frailty there [32]. Participants with incomplete data for the RISC were excluded from this analysis. Those declining to participate were also excluded. Ethical approval for this secondary analysis of the original dataset was obtained from the Galway University Hospitals ethics committee (LREC) in March 2019 (reference C.A 1429). Those who provided informed written consent were selected from all those recruited. Verbal assent was sought as an alternative, and if available, an identified next-of-kin was informed in cases in which the principle investigator indicated that the person lacked the capacity to give consent. Most of those screened but excluded from this study (*n* = 87) had incomplete data on the *RISC*. A further 27 were excluded because they were lost to follow-up (*n* = 10), were decompensated prior to or during the assessment (*n* = 7), declined to participate (*n* = 5), were unable to participate due to subsequent medical decompensation (*n* = 3), or were non-English speaking (*n* = 2). An a priori power calculation estimated a sample size of 275 was required [20]. 

### 2.2. Measures

#### 2.2.1. The RISC

The RISC is a risk-prediction instrument comprising three separate Likert scales called *Global RISC* scores, each ranking an individual’s one-year risk of an outcome from minimal (score of one) to extreme (score of five). The three outcomes assessed are institutionalisation, death, and hospitalisation. *Global RISC* scores are assessed individually using information recorded in the *RISC* score sheet (see Supplementary Materials). In this study, hospitalisation was taken as risk of 30-day readmission (dichotomised into yes/no) [33]. The three RISC scores for each of these three outcomes are scored individually based on the information recorded in the RISC score sheet (see Supplementary Materials), which includes demographic details and four domains of risk: Mental State, ADLs, Medical State, and Other. Each of these domains is scored across three individual, collapsing (if scored as “no”, the rater proceeds to the next domain), and sequential steps as follows. Step 1 assesses if a concern for a domain is present. If it is, the rater proceeds to Step 2 to assess its severity (as mild, moderate, or severe). The rater then completes Step 3, an assessment of the individual’s Caregiver Network, a composite of all known available formal and informal supports [34]. This is also scored from one (the network can manage the concern) to five (the network is absent but needed or a liability, e.g., abusive). Each step contributes to the subjective decision of the rater to score the three RISC scores. For this analysis, all three scores were added together to provide an additional, novel Overall RISC score.

#### 2.2.2. Other Screens

In this analysis, the RISC was compared with three other risk-prediction and/or frailty screens, the CFS [30], the Identification of Seniors At Risk (ISAR) [35], and the Programme of Research to Integrate Services for the Maintenance of Autonomy 7 (PRISMA-7) [36]. The CFS is a short, nine-point clinical measure of frailty that is used to identify and stratify patients after assessment. It has been validated in multiple studies in different hospital settings as a measure of frailty [20,37,38]. The CFS provides written descriptions identifying patients as being very fit (score of one) through to terminally ill (score of nine). Those scoring four are considered very mildly frail or pre-frail, whereas those graded between five (mild) and eight inclusive (very severely) are categorised as frail. The CFS can be adjusted for dementia based on the stage of functional impairment [30]. The PRISMA-7 is a seven-item questionnaire that asks questions related to age, gender, general health, social support, and activities of daily living [36]. A single point is scored for each with a cut-off score of ≥3 indicating that there is a risk of frailty and adverse outcomes including mortality [20]. The ISAR is a six-item screen scored from 0 to 6 (maximum score), which includes questions on care requirements, number of recent hospitalisations and illnesses, eye sight, memory, and polypharmacy (three of more medications). Those patients scoring ≥2 are classified as being at a high risk of adverse outcomes and are more likely to be frail [17,22,35]. Although the ISAR was created to measure risk rather than frailty—which, although related, are distinct concepts [23,24]—it has high sensitivity for frailty in the ED [17]. It has fair diagnostic accuracy in predicting specific adverse healthcare outcomes such as death in this setting [39,40].

### 2.3. Experimental Design

Prior to the study, research assistants (qualified doctors in training working in geriatric medicine) received education on the study protocol and instruments used (described in detail below). All triage nurses working in ED received information on frailty and were trained to score the screening instruments selected for the study. Inter-rater reliability (IRR) testing was conducted with the nurses, targeting a correlation coefficient of >0.6 based on three case studies and, once the study commenced, on a small sample (*n* = 20) of patients. The IRR for the latter was greater than 0.6 for all screening instruments (range from 0.62 to 0.78) [20]. Data were collected over two weeks in February–March 2016 as part of a quality improvement program in preparation for the introduction of a dedicated inpatient frailty service. To provide context, in 2016, the year of data collection, there were 64,096 recorded attendances to the ED studied. Of these, 9407 attendees were aged ≥70 (14.7% of all attendees). 

Study-specific screening was performed in the ED after the standard ED triage was completed (routine care), in cases in which it was possible, 24 h/day, Monday to Sunday inclusive. Although no longer the case, during the period of data collection, all patients who were referred to the acute medical assessment unit were initially triaged in ED. Screening was conducted after the standard ED triage was completed (routine care). Test scores of all three instruments were then placed aside and concealed from the research team (in a sealed box). Triage nurses were blind to the reference standard for determining if patients were frail, i.e., the CGA. Research assistants then reviewed patients’ medical records and medication lists and scored a battery of standardised assessments including the *RISC*. In cases in which a collateral history was available, it was obtained, and carers completed a Caregiver Burden Score (CBS). Within 24 hrs, patients were assessed by a consultant geriatrician who completed a CGA supported by the information obtained by the research team and who was blind to the screening test scores, i.e., the screens were not used to support the frailty classification. The consultant adjudicated for all cases to ensure quality control. Attendees not requiring admission were prioritised for CGA before discharge home. In cases in which patients were discharged before this was completed, a (modified) telephone assessment was conducted. 

Whether persons were frail or non-frail was determined independently by a consultant geriatrician using information available from the CGA. The following variables were collected as part of the CGA: age (date of birth), sex, history of presenting complaint, past medical history, MTS score, and current medication list. Polypharmacy was defined as the receipt of ≥4 medications. This definition was selected for convenience, as it is included as a question (Do you take 4 of more different types of medicine?) in one the frailty assessment instruments, the Groningen Frailty Indicator (GFI) [41]. Furthermore, it is a common cut-off for polypharmacy in the medical literature [42]. The following instruments were also collected as part of the CGA: the FRAIL Scale and the GFI are frailty-specific assessments. The FRAIL scale asks five questions related to the physical features of frailty, such as the presence of weight loss and fatigue, and is scored from 0 (not frail) to 5 (most frail) [43]. It was used specifically to identify pre-frailty: scores of 1 or 2/5 suggest pre-frailty, and scores >2 suggest frailty [43]. The GFI is a 15-point yes/no questionnaire exploring physical, cognitive, social, and psychological frailty. It was used to evaluate frailty domains, with a cut-off of ≥4/15 identifying moderate–severe frailty [41]. Other components of the CGA included assessments and questionnaires examining multi-morbidity, nutrition, cognition, measures of quality of life (QOL), and caregiver strain. Multi-morbidity was defined as the presence of ≥2 conditions [44] and was measured with the Charlson Co-morbidity Index (CCI); scores ≥5 indicate high mortality [45]. Nutritional status of patients was based on a measurement of body mass index (BMI) and an assessment of nourishment from the Mini-Nutritional Assessment Short Form (MNA-SF) [46], taking a cut-off of ≤11 for risk of malnutrition [47]. Cognitive impairment was identified using the Alzheimer’s Disease 8 (AD8) [48,49] and the Abbreviated Mental Test Score (AMTS) [50]. If a collateral history was unavailable, the patient was asked to complete the participant-rated version of the AD8 (pAD8) [49]. A cut-off of ≥2 on the AD8/pAD8 and <7/10 on the AMTS were used. Patient QOL was determined using the Euroqol EQ-5D, including its Visual Analogue Scale (VAS) [51] and the Short-Form (SF-36) [52]. The VAS is scored from 0 (worst imaginable) to 100 (best imaginable health state today) [51]. The SF-36 measured General Self-Rated Health (GSRH), determined from a single question: “In general, would you say your health is, excellent; very good; good; fair or poor?” [52]. Caregiver strain or burden was scored using the CBS [53]. Adapted from the Zarit Burden Interview, the CBS is composed of six questions asking carers or close family to rate how (if required) their role as caregiver affects their own lives [53]. It is scored from zero (never) to six (always) to a total score of 30, with scores ≥15/30 suggesting burden and scores ≥25/30 indicating burnout [53]. 

The outcome measures recorded and used in this analysis were one-year mortality, one-year rate of institutionalisation, and three different hospitalisation outcomes: 30-day re-attendance rate if discharged from the ED, total LOS, and 30-day readmission rate, if admitted. Data were obtained from the hospital’s Patient Administration System (PAS) and from the county’s local placement forum (LPF). Re-admission to hospital was defined as any admission to an acute hospital in the hospital healthcare group, excluding attendances in the ED that did not result in admission. Data for hospitals outside this group were not available, as they are not linked to the hospital’s PAS. Institutionalisation was defined as admission to a nursing home (providing low or high levels of ADL dependency) excluding those living in sheltered accommodation (i.e., assisted living/supportive housing programmes or in retirement communities). Discussion and approval for long-term care at the LPF was regarded as institutionalisation, as the exact date of admission was often unknown unless patients were discharged to nursing homes directly from hospital. Most outcomes were recorded as dichotomised variables, except for LOS (continuous variable measured in days). Date of death was also obtained from the PAS.

### 2.4. Statistical Analysis 

Data were analysed with SPSS V24.0 (Chicago, IL, USA) and R version 3.5.0 (23 April 2018)—”Joy in Playing” (R Core Team, 2018). The Shapiro–Wilk test and quantile–quantile (Q-Q) plots assessed the normality of data, finding that the majority had a non-normal distribution. Pearson’s Correlation assessed IRR. The Mann–Whitney U test was used to compare samples. The Chi-squared test or Fishers exact test was used to compare frequencies from contingency tables for categorical variables. The sensitivity, specificity, positive predictive value (PPV), and negative predictive value (NPV) of each instrument were calculated at different cut-offs. Accuracy was determined from the area under the receiver operating characteristic curves (ROC). These were then compared using the DeLong approach [54]. Optimal cut-off scores were produced from Youden’s Index [55]. This cut-off point optimises the test’s ability to differentiate outcomes when equal weight is applied to the sensitivity and specificity [55]. Kaplan–Meier Survival Analysis was used to examine the proportion surviving to the selected end-point, one-year, which is the prediction time for the *RISC* tool. Time to event was defined in days from the date of assessment to the date of death, and survival curves were plotted. Patients still alive at one-year were right-censored at the time in days from baseline. The log-rank test was used to test the equality of the survival functions weighting all points in time equally, comparing survival times between those scored as being at a high risk of death versus those scored as low-risk of death based on their *RISC* classification (dichotomised based on high- and low-risk *RISC* scores at defined cut-offs, i.e., to assess outcomes by *RISC* score level—which is usually scored from 1 to 5, low to high—values were collapsed into low- (1:2 = “low”) versus high- (3:5 = “high”) grade risk. This study complies with the Standards for Reporting of Diagnostic Accuracy (STARD) guidelines [56], see Supplementary Materials.

## 3. Results

### 3.1. Characteristics

**Figure 1:**
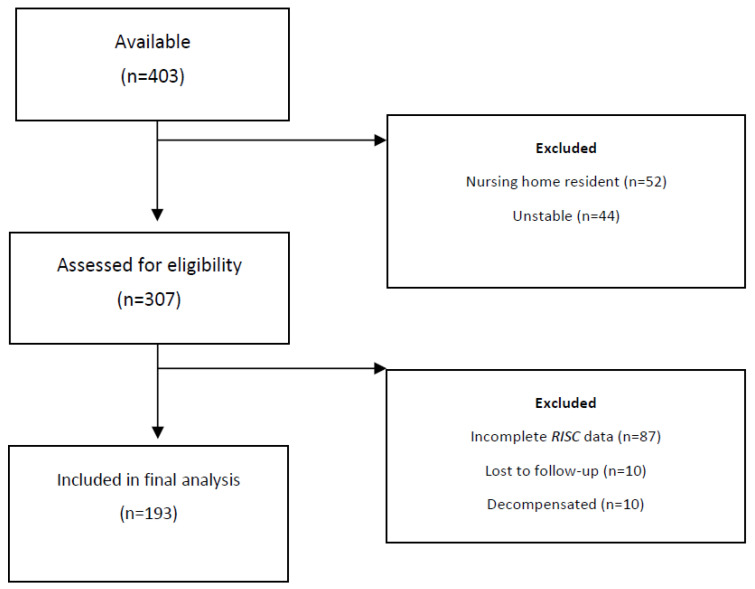
Flow diagram detailing patient selection.

In total, 307 patients aged ≥70 years were available for screening over the two weeks of the study. These represented 76% (307/403) of all those aged ≥70 attending the ED during this period. The remaining patients (*n* = 96) were not screened, either because they were nursing home residents or were deemed unsuitable because they scored an MTS score of one (implying they required urgent triage or were immediately transferred to the resuscitation area in ED). Of those available, a total of 193 patients (63%) were included in this analysis. These are presented in a flow diagram (Figure 1). 

The median age (interquartile range—IQR) of the patients included (*n* = 193) was 79 (83−74 = ±9) years, of which most (55%) were female. Polypharmacy (≥4 medications) was common amongst those included (81%). The median MNA-SF nutritional score was 11 ± 5. One-fifth (21%) reported that that their self-rated QOL was very good or excellent (GSRH); the median EQ-5D score was 60 ± 38. Co-morbidity was common, as reflected by a median CCI score of 5 ± 2. Similarly, a high proportion likely had cognitive impairment, with 26% scoring ≥2 on the AD8. Based on the CGA, the majority, (60%), were found to be frail. Those that were recorded as frail were statistically significantly older (*p* = 0.03) and had lower median MNA-SF (*p* < 0.001), AMTS (*p* = 0.05), GSRH (*p* < 0.001), and EQ-5D VAS (*p* < 0.001) scores than non-frail patients. They had higher median AD8 scores (*p* < 0.001), suggesting higher levels of cognitive impairment with a significantly greater proportion of those identified as frail scoring ≥2 on the AD8 (40% versus 6.5%, *p* < 0.001). They also had higher CBS scores, as reported by a collateral history, where it was available (*p* < 0.001), indicating higher levels of caregiver strain (care burden). There were no statistically significant differences level of co-morbidity based on the CCI or sex between frail and non-frail patients. Patient characteristics are provided in Table 1 according to their frailty status. 

The median values for all three *Global RISC* scores showed that most participants were classified as being at lower (one-year) risk of each of the three adverse outcomes: hospitalisation, institutionalisation, and death. Dichotomising patients into being at a low risk (*RISC* scores of 1 and 2) or high risk (*RISC* scores of 3, 4, and 5) showed that most older adults attending ED were considered to be at a high risk of further hospitalisation (64%), but fewer were predicted to have a high risk of institutionalisation (22%) or death (35%) within the next year. Frail patients had statistically significantly higher median *RISC* scores.

### 3.2. Adverse Outcomes

In total, the ED conversion rate (proportion admitted from ED to hospital) was 77%. Dichotomising the *Global RISC* for each outcome into low- and high-risk categories showed that those with higher *RISC* scores for hospitalisation (*p* = 0.018) and death (*p* = 0.015) were significantly more likely to be admitted. For those admitted, the median LOS was 8 ± 9 days, with 58% having a prolonged LOS (≥7 days). Those with higher *RISC* scores had significantly prolonged admissions. Of those admitted, 20% were re-admitted within 30 days of discharge. Again, patients with higher *Global RISC* scores were more likely to be re-admitted. At one-year follow-up, 13.5% of patients were admitted to a nursing home (including those for whom the decision to be admitted was formalised after approval at the local placement forum). Those with higher *RISC* scores were more likely to be institutionalised. The one-year mortality rate was 17%. Irrespective of *RISC* score, the mortality rate was statistically significantly higher amongst those classed as being at a high risk: 34% of those who scored as being at a high risk on the *Global RISC* score for death died at one year compared to 8% of those who scored as being at a lower risk, *p* < 0.001. The comparison between patients scored as being at a high risk and those scored as being at a low risk on each of the three *RISC* domains (hospitalisation, institutionalisation, and death) is presented in Table 2.

### 3.3. Accuracy

**Figure 2:**
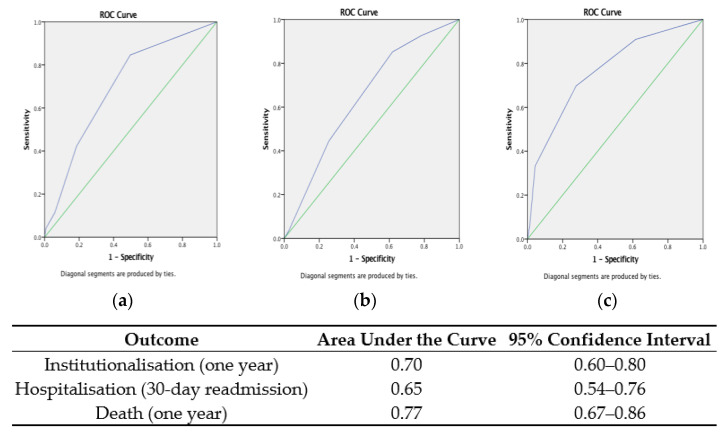
Receiver operating characteristic curves showing the accuracy of the Global Risk Instrument for Screening in the Community (*RISC*) in predicting one-year (**a**) institutionalisation, (**b**) hospitalisation, and (**c**) death.

**Figure 3:**
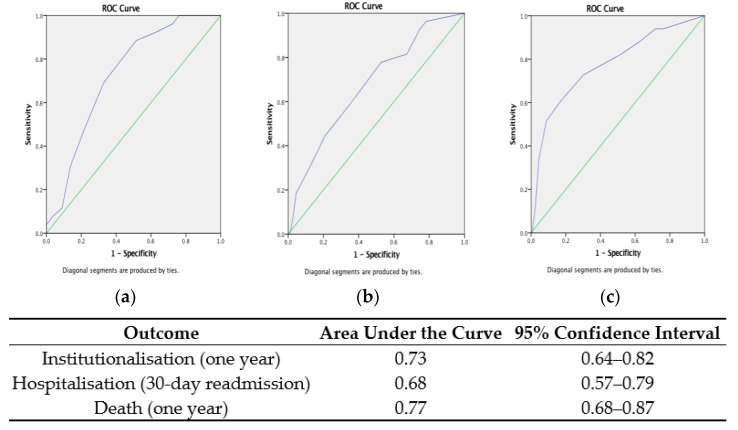
Receiver operating characteristic curves showing the accuracy of the pooled *Overall* Risk Instrument for Screening in the Community (*RISC*) score in predicting one-year (**a**) institutionalisation, (**b**) hospitalisation, and (**c**) death.

Examining the accuracy of the *Global RISC* for each corresponding outcome showed that the *RISC* was most accurate for predicting mortality and nursing home admission at one year: AUC 0.77 (95% CI: 0.67–0.86) and 0.70 (95% CI: 0.60–0.80), respectively. The *RISC* score for hospitalisation had the lowest predictive accuracy for hospital re-admission at 30 days: AUC 0.65 (95% CI: 0.54–0.76). ROC curves with the AUC for each of the three *Global RISC* scores for their corresponding outcomes are presented in Figure 2. Pooling the *RISC* scores for each of the *Global RISC* domains (hospitalisation, institutionalisation, and death) to produce a combined *Overall RISC* score gave a median *Overall RISC* score of 7 (IQR 8−5 = ±3) out of a total combined score of 15 points. The accuracy of the new *Overall RISC* and ROC curves for one-year mortality and institutionalisation and 30-day readmission are presented in Figure 3.

**Figure 4:**
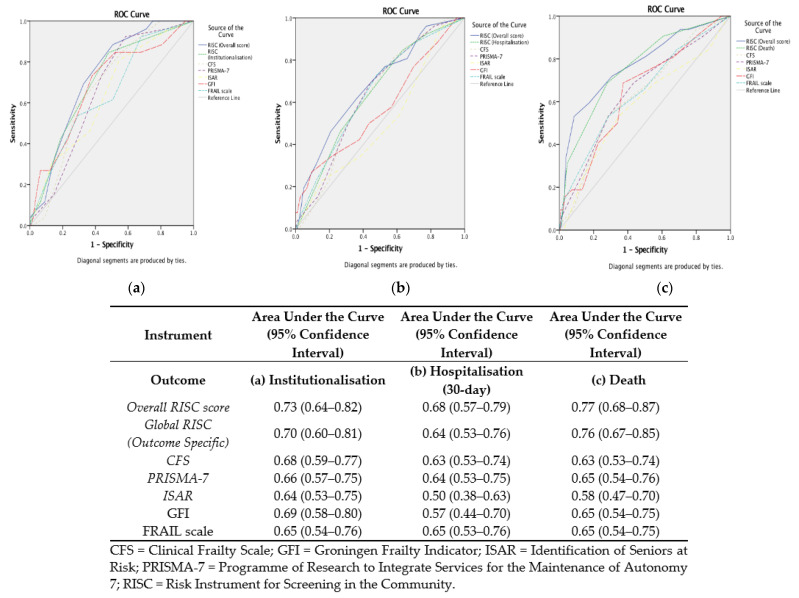
Receiver operating characteristic curve comparing the accuracy of each screen for (**a**) institutionalisation, (**b**) hospitalisation at 30 days, and (**c**) mortality at one year from assessment.

Comparing the accuracy of the individual *RISC* scores and the other screens (CFS, ISAR, and PRISMA-7) and frailty assessment instruments (FRAIL scale and GFI) showed that the *Overall RISC* score had the highest AUC values for each outcome, albeit the *RISC* scores did not have greater statistical significance over the other instruments. These are presented as ROC curves in Figure 4. None of the instruments were useful for predicting which older adults were re-admitted within 30 days. The sensitivity, specificity, NPV, PPV, and false positive and negative rates for the *Overall RISC* score for death for a range of cut-off points are presented in Table 3. The optimal cut-off identified for predicting one-year risk of death, using Youden’s Index to select the cut-off point, was ≥9/15, which gave a modest sensitivity of 61% and a high specificity of 83%. Reducing the cut-off score increased the sensitivity but yielded a lower specificity. A cut-off of ≥8/15 arguably gave the best balance between sensitivity and specificity (73% and 70%, respectively). The PPV for the *Overall RISC* score for mortality was low–moderate (43% at a cut-off of ≥9/15), irrespective of the cut-off selected. The false positive rate for mortality was high (57% at a cut-off of ≥9/15). The *Overall RISC* score had good diagnostic accuracy for frailty, AUC of 0.84, 95% CI: 0.78–0.89). It had a moderate sensitivity of 77% and specificity of 74% at an optimal cut-off of ≥7/15 for separating those who were categorised as frail versus those scored as non-frail using the CGA.

### 3.4. Survival Analysis

**Figure 5:**
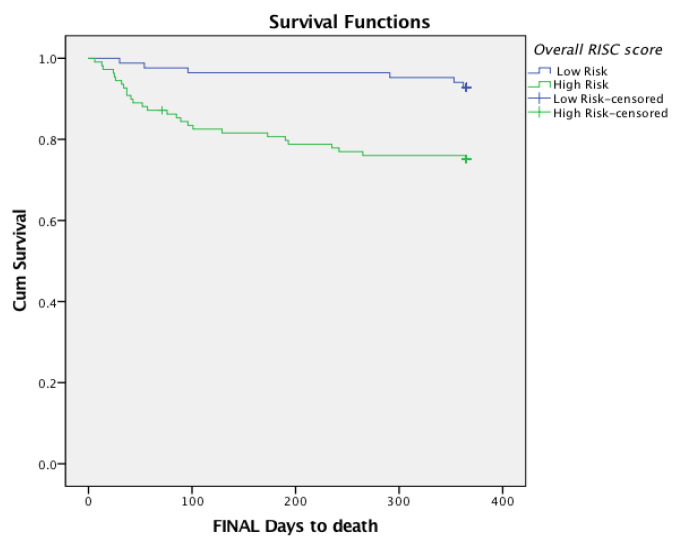
One-year survival according to the combined *Overall RISC* score.

At one-year follow-up, patients who had been identified as frail had reduced survival times compared to non-frail individuals (Log-rank X^2^(1) = 10.5, *p* = 0.001). An examination of survival according to the *RISC* score revealed that 25% of those who had been scored as being at a high risk on the combined *Overall RISC* score had died compared to just 7% of individuals who had been scored as being at a low risk; 27/33 (82%) of deaths at one-year occurred in those who had been classified as being at a high risk. Those screening high-risk (scoring ≥ 7/15 on the *Overall RISC* score) were more likely to have died at one-year than those screening low-risk (*p* = 0.001) and had statistically significantly reduced survival times (Log-rank X^2^(1) = 10.7, *p* = 0.001) compared to those screening as low-risk on the combined *Overall RISC* score (Figure 5).

## 4. Discussion

This study presents a clinimetric evaluation of the *RISC*, a short, global, subjective risk-prediction instrument for adverse healthcare outcomes in an older sample attending a large university hospital ED in the West of Ireland. All those included were aged ≥70, medically stable (based on an MTS score >1) and community-dwelling (i.e., nursing home residents were excluded). The study also compares the diagnostic accuracy of the *RISC* to a selection of brief, commonly used, frailty-screening assessment and risk-prediction instruments but in an ED, rendering this study the first validation of the *RISC* in this setting, where such testing remains under-researched [57]. It compares the established *RISC* and a new metric, the combined *Overall RISC* score (merging each of the three *Global RISC* scores for institutionalisation, hospitalisation, and death), to identify a patient’s frailty status based on an independent CGA. 

The results suggest that the *RISC* (*Global RISC*) scores for death and institutionalisation have the highest diagnostic accuracy in identifying one-year mortality and nursing home admission, respectively. These, however, were not statistically significantly better than the other instruments examined. Likewise, the *Overall RISC* score, combining all three *RISC* (*Global RISC*) scores, had slightly higher but not significantly greater diagnostic accuracy for any of the adverse healthcare outcomes investigated. At best, the short screens examined had fair diagnostic accuracy with an AUC of ≤0.80. None of the instruments examined were able to predict 30-day hospital readmission. All had poor to no diagnostic accuracy (AUC ranging between 0.50 and 0.68). This was expected, as most hospital readmission risk-prediction models have poor diagnostic accuracy [58]. Diagnostic accuracy was highest for mortality (c statistic range 0.58–0.77). This is in keeping with a recent systematic review showing that most prognostic indices for death have c-statistics ≥0.70, though none have excellent accuracy (≥0.90) [59]. These results mirror a recent systematic review which showed that short risk-prediction instruments including the *RISC* have poor accuracy in predicting functional decline, hospitalisation, institutionalisation, and death amongst community-dwelling older adults [23]. It also supports two more recent systematic reviews examining the use of the ISAR instrument in the ED, which showed mixed results in different studies, with, at best, modest predictive accuracy for a range of adverse outcomes including readmission and mortality [39,40]. 

The other clinimetric properties of the combined *Overall RISC* (i.e., sensitivity, specificity, PPV, NPV, false positive/negative rates) were generally modest. The *Overall RISC* score yielded a modest sensitivity (61%) but high specificity (83%) for one-year risk of death based on the cut-off identified as optimal using Youden’s Index (≥9/15). At this cut-off point, the PPV was 43% (95% CI: 29–58%). PPV is particularly important for a screening instrument, i.e., the ability of a test to identify true positives [60]. Altering the stringency of the test by lowering the cut-off to ≥7/15 increased sensitivity to 82% but provided a specificity of only 48%. Applying this cut-off would, in principle, render it useful for screening. Furthermore, although high sensitivity is important for a screening test, specificity is also important for reducing the need for further and extensive diagnostic assessment [61]. At a cut-off of ≥7/15, the false positive rate would increase to 75%. Although screening does not remove the need for a diagnostic next step, a low specificity and a very high false positive rate would mean that in practice, almost all patients would have to automatically progress to diagnostic testing, negating the need for screening. 

These results, which are highlighted by the example of one-year mortality, suggest that the *Overall RISC* score is a relatively poor screening instrument for adverse outcomes in ED. Although a lower cut-off would improve sensitivity, this would result in a lower PPV and a high false positive rate, creating redundancy and adding extra work (i.e., extra costs and annoyance) [60] to any routine risk-prediction in already busy EDs. Given the low levels of diagnostic accuracy of the other instruments examined and the results of multiple systematic reviews examining risk-prediction instruments in settings other than ED, it is reasonable to conclude that their use should be based on other features, such as time constraints and acceptability (to staff and patients), for which risk-stratification to tailored treatments, particularly limited resources (e.g., admission to a geriatric specialist ward) is desirable. This study shows that the *RISC* compares favourably with the other short screens tested. Given that the median administration time of the *RISC* at <5 min (close to one minute when only the Likert scales are applied) is similar to and often shorter than the three frailty screens studied [27], and given that it is acceptable to a wide variety of healthcare professionals [28], it is reasonable to consider it favourably in selecting an instrument to correctly identify frailty in the ED [25]. However, the other clinimetric properties of the *Overall RISC* for detecting frailty were more modest. Based on Youden’s Index, for the optimal cut-off (≥7/15), the sensitivity and specificity were 77% and 74%, respectively. In addition, the prevalence of frailty at 60% was high, which affects the healthcare professional’s ability to interpret the PPV and NPV. For screening instruments, high prevalence can lower specificity [62] and lead to possible spectrum bias whereby performance is altered by the settings, i.e., artificially high levels of frailty in ED are not likely to be generalisable outside of this environment [63]. 

This study has a number of strengths and limitations. A strength is the use of CGA to identify frailty. The approach used was broad and completed by a consultant geriatrician taking a multi-domain approach to diagnosing frailty supported by a range of assessment scales. Follow-up of adverse events was conducted thoroughly using the best available healthcare records. There are several limitations. The generalisability of the results is limited by the homogenous nature of the sample (i.e., community-dwelling older adults in the West of Ireland attending a single ED) and the restricted sampling time frame (two weeks in Spring 2016). Nursing home residents were excluded, potentially reducing generalisability. These comprise a large proportion of presentations to ED and have higher rates of admission and adverse events [20,64]. Given the expectation that these are all high-risk cases, a pragmatic decision was made to exclude nursing home patients and prioritise the inclusion of those for whom the risk is less clear. To minimise selection bias, consecutive screening was used to achieve a representative sample, and every attempt was made to ensure screening continued outside of daytime shift hours. Despite this, selection bias may still have occurred; during busy triage periods or during acute emergencies, especially during the on-call night hours when staffing levels were lower, some patients who may otherwise have been suitable were not screened. No data on those not screened were available. Furthermore, many potentially suitable patients who were recruited mainly during on-call hours were missing elements from their CGA or did not have it completed. In addition, some patients attending ED during the night but not requiring admission, despite being screened at triage, were lost to follow-up. These challenges are reflected in the missing screening and assessment data, in that some but not all scores were completed for each individual. This was anticipated in advance and accounted for in the sample size calculation, and an attempt was made to follow up all patients who had been screened at triage in cases in which it was possible (e.g., on the wards). Finally, the outcomes chosen (design-related bias) could have influenced the results of the diagnostic accuracy testing, e.g., restricting 30-day readmission rates only to those who were admitted could have influenced and potentially lowered the predictive validity of the instruments. 

## 5. Conclusions

The results suggest that practitioners should be aware that short screens for frailty in ED are useful in supporting the clinical identification of frail individuals rather than patients who are likely to develop adverse healthcare outcomes. Furthermore, the *RISC* has clinimetric properties that are similar to more commonly used short risk-prediction or frailty screening instruments and can be used to identify vulnerable and frail patients in ED as part of a two-step screening and assessment diagnostic pathway. Nevertheless, until additional research is completed, the decision to choose one over another is contingent on the personal preference of staff and ED service requirements. Further research validating the *RISC* in other settings such as hospital wards, intensive care, rehabilitation units, primary care, and nursing homes is recommended to examine its diagnostic accuracy and confirm its use as a broad but simple measure of risk of adverse outcomes among older people. It is also important to compare the *RISC* with other short frailty screens in these settings to confirm the external validity of the instrument. Investigating the *RISC* with other combinations of existing brief instruments for use in the ED as part of a two-step diagnostic pathway to maximise sensitivity and specificity may also be useful. 

In summary, this study, which examines the *RISC* subtests (components) and the combined *Overall RISC* score in ED, shows that this instrument has good and comparable accuracy to more established risk-prediction instruments to identify three adverse outcomes in acute care (institutionalisation, hospitalisation, and death). This suggests that a risk-prediction instrument that was originally designed for stratifying risk in a community setting is also useful across the interface between acute and community healthcare services. Given that this instrument is quick to administer and could be used over time to monitor risk levels across settings and transitions of care, this study suggests that the *RISC* can help identify patients who are more likely to experience such adverse events and, hence, those in need of tailored interventions. However, further assessment is required with larger samples to externally validate these findings. 

## Figures and Tables

**Table 1:** Characteristics of included patients (*n* = 193) according to frailty status based on the comprehensive geriatric assessment.

Predictor	Total (*n* = 193) Median (Q3 − Q1 = ±IQR) or %	Frail (*n* = 116) Median (Q3 − Q1 = ±IQR) or %	Non Frail (*n* = 77) Median (Q3−Q1 = ±IQR) or %	*p* = X
Age (Years)	79 (83 − 74 = ±9)	80 (84 − 75 = ±9)	77 (82 − 73 = ±9)	z = −2.1 *p* = 0.03 *
Sex (% Female)	55%	53%	58%	X^2^ (1) = 0.47 *p* = 0.49
Polypharmacy (% ≥4 Medications)	81%	92%	65%	X^2^ (1) = 21.6 *p* < 0.001 *
BMI ** (Kg/M^2^)	26 (29 − 22 = ±7)	25 (27 − 21 = ±6)	26 (29 − 23 = ±6)	z = −1.4, *p* = 0.15 t = 1.1, *p* = 0.28 **
CCI	5 (7 − 5 = ±2)	6 (8 − 5 = ±3)	5 (6 − 4 = ±2)	z = −4.3 *p* < 0.001 *
MNA-SF	11 (13 − 8 = ±5)	9 (12 − 7 = ±5)	12 (14 − 10 = ±4)	z = −5.8 *p* < 0.001 *
AD8	0 (2 − 0 = ±2)	1 (3 − 0 = ±3)	0 (0 − 0 = ±0)	z = −5.8 *p* < 0.001 *
Cognitive Impairment (%) ***	26%	40%	6.5%	X^2^ (1) = 26 *p* < 0.001 *
AMTS	9 (10 − 7 = ±3)	9 (10 − 5 = ±5)	10 (10 − 8 = ±2)	z = −1.9 *p* = 0.05
CBS	4 (17 − 0 = ±17)	12 (19 − 3 = ±16)	0 (0 − 0 = ±0)	z = −4.5 *p* < 0.001 *
EQ-5D (VAS)	60 (80 − 42 = ±38)	50 (60 − 35 = ±25)	80 (85 − 60 = ±25)	z = −6.5 *p* < 0.001 *
GSRH (% Very good /excellent)	21%	4.5%	44%	X^2^ (1) = 43.5 *p* < 0.001 *
GFI	4 (7 − 2 = ±5)	6 (7 − 4 = ±3)	2 (3 − 1 = ±2)	z = −10.5 *p* < 0.001 *
FRAIL scale	2 (3 − 0 = ±3)	3 (3 − 1 = ±2)	0 (1 − 0 = ±1)	z = −8.7 *p* < 0.001 *
ISAR	3 (4 − 2 = ±2)	4 (4 − 3 = ±1)	2 (3 − 1 = ±2)	z = −6.9 *p* < 0.001 *
CFS	4 (5 − 3 = ±2)	5 (6 − 4 = ±2)	3 (4 − 2 = ±2)	z = −7.7 *p* < 0.001 *
PRISMA-7	3 (5 − 2 = ±3)	5 (6 − 3 = ±3)	2 (3 − 1 = ±2)	z = −8.8 *p* < 0.001 *
*RISC* Hospitalisation	3 (4 − 2 = ±2)	3 (4 − 3 = ±1)	2 (3 − 1 = ±2)	z = −7.9 *p* < 0.001 *
*RISC* Institutionalisation	2 (2 − 1 = ±1)	2 (3 − 1 = ±2)	1 (2 − 1 = ±1)	z = −6.2 *p* < 0.001 *
*RISC* Death	2 (3 − 1 = ±2)	2 (3 − 2 = ±1)	1 (2 − 1 = ±1)	z = −6.4 *p* < 0.001 *

AD8—Alzheimer’s Disease 8; AMTS—Abbreviated Mental Test Score; BMI—Body Mass Index; CBS—Caregiver Burden Score; CCI—Charlson Co-morbidity Index; CFS—Clinical Frailty Scale; EQ-5D-VAS—Euroqol EQ-5D Visual Analogue Scale; GSRH—General Self-Rated Health; GFI—Groningen Frailty Indicator; ISAR—Identification of Seniors at Risk; MNA-SF—Mini-Nutritional Assessment Short Form; PRISMA-7—Programme of Research to Integrate Services for the Maintenance of Autonomy 7; RISC—Risk Instrument for Screening in the Community. * Statistically significant; ** BMI is normally distributed: also presented as mean and standard deviation (SD); *** Based on an AD8 cut-off of ≥2, suggesting cognitive impairment.

**Table 2:** Comparison of each *Global* Risk Instrument for Screening in the Community (*RISC*) status, high versus low risk, by adverse outcomes (* statistically significant).

Outcome	Total	*RISC* Hospitalisation (High) (*n* = 123)	*RISC* Hospitalisation (Low) (*n* = 70)	*p* = x	*RISC* Institutionalisation (High) (*n* = 42)	*RISC* Institutionalisation (low) (*n* = 151)	*p* = x	*RISC* Death (High) (*n* = 68)	*RISC* Death (Low) (*n* = 125)	*p* = x
ED Conversion Rate (% admitted)	77%	82%	67%	X^2^(1) = 5.6 *p* = 0.018 *	79%	76%	X^2^(1) = 0.1 *p* = 0.74	87%	71%	X^2^(1) = 6.0 *p* = 0.015 *
LOS (Median days ± IQR)	8 (15 − 4 = ±9)	9 (17 − 5 = ±12)	5 (10 − 3 = ±7)	z = −2.7 *p* = 0.007 *	12 (22 − 7 = ±15)	7 (14 − 4 = ±10)	z = −2.8 *p* = 0.005 *	10 (18 − 5 = ±13)	6 (12 − 4 = ±8)	z = −2.7 *p* = 0.007 *
LOS (% ≥7 days)	58%	65%	43%	X^2^(1) = 6.8 *p* = 0.009 *	79%	52%	X^2^(1) = 7.5 *p* = 0.006 *	71%	49%	X^2^(1) = 6.9 *p* = 0.009 *
Hospitalisation (readmission ≤ 30 days)	20%	25%	9%	X^2^(1) = 5.3 *p* = 0.02 *	36%	16%	X^2^(1) = 5.7 *p* = 0.02 *	30%	14%	X^2^(1) = 5.3 *p* = 0.02 *
Nursing Home Admission (%)	13.5%	19%	4%	X^2^(1) = 8.0 *p* = 0.005 *	26%	10%	X^2^(1) = 7.5 *p* = 0.006 *	24%	8%	X^2^(1) = 9.1 *p* = 0.003 *
Death (within 1 year)	17%	21%	10%	X^2^(1) = 4 *p* < 0.046 *	31%	13%	X^2^(1) = 7.2 *p* = 0.007 *	34%	8%	X^2^(1) = 21 *p* < 0.001 *

ED = emergency department; IQR = interquartile range; LOS = length of stay.

**Table 3:** Sensitivity, specificity, positive predictive value (PPV), and negative predictive value (NPV), with 95% confidence intervals (CI), for the *Overall RISC* (Risk Instrument for Screening in the Community) score for one-year mortality. * Optimal cut-off based on Youden’s Index.

*Overall RISC* Cut-off Score (Range 3–12)	Youden’s Index (J)	Sensitivity (95% CI)	Specificity (95% CI)	False Negative (95% CI)	False Positive (95% CI)	NPV (95% CI)	PPV (95% CI)
≥4	0.18	94% (78–89)	24% (18–31)	5% (1–18)	80% (72–86)	95% (82–99)	20% (14–28)
≥5	0.22	94% (78–89)	28% (22–36)	4% (1–16)	79% (71–85)	96% (84–99)	21% (15–29)
≥6	0.26	88% (71–96)	62% (30–46)	6% (2–16)	77% (69–84)	77% (84–98)	23% (16–31)
≥7	0.31	82% (64–92)	48% (41–57)	7% (3–15)	75% (66–83)	93% (85–97)	25% (17–34)
≥8	0.43	73% (54–86)	70% (62–77)	8% (4–14)	67% (54–77)	93% (86–96)	33% (23–46)
≥9 *	0.44	61% (42–77)	83% (76–88)	9% (5–15)	57% (42–71)	91% (85–95)	43% (29–58)
≥10	0.42	51% (44–69)	91% (85–95)	10% (6–16)	45% (28–64)	90% (84–94)	55% (36–72)
≥11	0.29	33% (19–52)	96 (91–98)	13% (8–19)	39% (18–64)	87% (81–92)	61% (36–82)
≥12	0.09	12% (4–29)	97% (93–99)	16% (11–22)	50% (17–83)	84% (78–89)	50% (17–83)

## Data Availability

On request from the corresponding author.

## Supplementary Materials

The following supporting information can be downloaded at: https://www.mdpi.com/article/10.3390/ijerph20043734/s1, Supplementary S1. The Risk Instrument for Screening in the Community (*RISC*) scoring sheet. © O’Caoimh & Molloy 2013. Supplementary S2. Standards for Reporting of Diagnostic Accuracy (STARD) Checklist.
