# Supplementary material for: Validation of the Risk Instrument for Screening in the Community (RISC) among Older Adults in the Emergency Department
Source: Int J Environ Res Public Health. 2023 Feb 20;20(4):3734. doi: 10.3390/ijerph20043734 (PMC9966437; doi:10.3390/ijerph20043734)
Supplement: Supplementary file 1 [file ijerph-20-03734-s001.zip › ijerph-2190692-supplementary.pdf]

Supplementary S1. The Risk Instrument for Screening in the Community (*RISC*) scoring sheet. © O'Caoimh & Molloy 2013. Available at: https://bmcgeriatr.biomedcentral.com/articles/10.1186/1471-2318-14-104 (Accessed on 1 December 2022)

RISC Score Sheet©

Demographics

Personal Details: **Name**______________________________

Address ______________________________
____
____

Gender : **M** ☐ **F** ☐ **DOB** / / **ID** ____________

Living Arrangements:

Alone ☐ **Spouse** ☐

Child ☐

Other ____________

**Instructions**	**Step 1**	**Step 2**	**Step 3**
**Domain**	**Concern**		**Caregiver Network**
If NO concern for a Domain, move on to the next Domain. Complete all 4 domains	Is there concern about issues in this domain? (Circle **Y**es or **N**o) Then complete Step 2	Circle the present severity of the concern (Circle:1,2,3) **1. Mild.** **2. Moderate.** **3. Severe.** Then complete Step 3	Is the caregiver network able to manage (Circle:1,2,3,4 or 5) **1.Can manage** **2.Carer strain** **3.Some gaps** **4.Cannot manage** **5.Absent/liability**
**1. Mental State**	**N Y** ↓ →	1 2 3	**1 2 3 4 5**
**2. ADLs**	**N Y** ↓ →	1 2 3	**1 2 3 4 5**
**3. Medical/Physical State**	**N Y** ↓ →	1 2 3	**1 2 3 4 5**
**4. Other specify**____________	**N Y** ↓ →	1 2 3	**1 2 3 4 5**

Global Risk Score

(circle 1,2,3,4 or 5)

**A. Institutionalisation** Overall risk of admission to long-term care {nursing home} in the next year.	**1** **Minimal / rare**	**2** **Low / unlikely**	**3** **Moderate / possible**	**4** **High / likely**	**5** **Extreme / certain**

**B. Hospitalisation** Risk of hospitalisation including prolonged admission or readmission in the next year.	**1** **Minimal / rare**	**2** **Low / unlikely**	**3** **Moderate / possible**	**4** **High / likely**	**5** **Extreme / certain**

**C. Death** Risk of death in the next year.	**1** **Minimal / rare**	**2** **Low / unlikely**	**3** **Moderate / possible**	**4** **High / likely**	**5** **Extreme / certain**

Global Risk Score Definitions

1. Minimal: Little or no serious consequence related to the risk / **Rare:** The event will almost never occur.
2. Low: Small impact from the risk, unlikely to cause serious harm / **Unlikely:** Low probability of the event occurring.
3. Moderate: Significant risk present / **Possible:** The event may occur but is infrequent or unlikely to occur soon.
4. High: Serious impact likely from the risk /**Likely:** High probability of the event occurring.
5. Extreme: Severe consequences likely /**Certain:** The event will almost certainly occur.

ADL = Activities of daily living including basic personal ADL (dressing, washing, walking) and instrumental ADL (shopping, cooking, using the telephone).

Supplementary S2. Standards for Reporting of Diagnostic Accuracy (STARD) Checklist

Section & Topic	No	Item	Reported on page # (N/A = Not applicable)
**TITLE OR ABSTRACT**			
	**1**	Identification as a study of diagnostic accuracy using at least one measure of accuracy<br>(such as sensitivity, specificity, predictive values, or AUC)	1
**ABSTRACT**			
	**2**	Structured summary of study design, methods, results, and conclusions<br>(for specific guidance, see STARD for Abstracts)	1
**INTRODUCTION**			
	**3**	Scientific and clinical background, including the intended use and clinical role of the index test	1-2
	**4**	Study objectives and hypotheses	2
**METHODS**			
Study design	**5**	Whether data collection was planned before the index test and reference standard were performed (prospective study) or after (retrospective study)	2-3 (prospective)
Participants	**6**	Eligibility criteria	2-3
	**7**	On what basis potentially eligible participants were identified<br>(such as symptoms, results from previous tests, inclusion in registry)	2-3
	**8**	Where and when potentially eligible participants were identified<br>(setting, location and dates)	2-3
	**9**	Whether participants formed a consecutive, random or convenience series	2 (consecutive)
Test methods	**10a**	Index test, in sufficient detail to allow replication	3
	**10b**	Reference standard, in sufficient detail to allow replication	3
	**11**	Rationale for choosing the reference standard (if alternatives exist)	N/A
	**12a**	Definition of and rationale for test positivity cut-offs or result categories<br>of the index test, distinguishing pre-specified from exploratory	3, 5
	**12b**	Definition of and rationale for test positivity cut-offs or result categories<br>of the reference standard, distinguishing pre-specified from exploratory	3
	**13a**	Whether clinical information and reference standard results were available<br>to the performers/readers of the index test	4 (blind to performers)
	**13b**	Whether clinical information and index test results were available<br>to the assessors of the reference standard	4 (blind to assessors)
Analysis	**14**	Methods for estimating or comparing measures of diagnostic accuracy	5
	**15**	How indeterminate index test or reference standard results were handled	5
	**16**	How missing data on the index test and reference standard were handled	3
	**17**	Any analyses of variability in diagnostic accuracy, distinguishing pre-specified from exploratory	N/A
	**18**	Intended sample size and how it was determined	3
**RESULTS**			
Participants	**19**	Flow of participants, using a diagram	6

	**20**	Baseline demographic and clinical characteristics of participants	6, 8 (Table 1)
	**21a**	Distribution of severity of disease in those with the target condition	8
	**21b**	Distribution of alternative diagnoses in those without the target condition	8
	**22**	Time interval and any clinical interventions between index test and reference standard	4
Test results	**23**	Cross tabulation of the index test results (or their distribution) by the results of the reference standard	8
	**24**	Estimates of diagnostic accuracy and their precision (such as 95% confidence intervals)	9-11
	**25**	Any adverse events from performing the index test or the reference standard	-
**DISCUSSION**			
	**26**	Study limitations, including sources of potential bias, statistical uncertainty, and generalisability	14
	**27**	Implications for practice, including the intended use and clinical role of the index test	14
**OTHER INFORMATION**			
	**28**	Registration number and name of registry	N/A
	**29**	Where the full study protocol can be accessed	On request from the authors
	**30**	Sources of funding and other support; role of funders	N/A
